# Composite Tissue Allotransplantation and Dysregulation in Tissue Repair and Regeneration: A Role for Mesenchymal Stem Cells

**DOI:** 10.3389/fimmu.2013.00188

**Published:** 2013-07-09

**Authors:** Anuja K. Antony, Katherine Rodby, Matthew K. Tobin, Megan I. O’Connor, Russell K. Pearl, Luisa A. DiPietro, Warren C. Breidenbach, Amelia M. Bartholomew

**Affiliations:** ^1^Department of Surgery, Division of Plastic, Reconstructive and Cosmetic Surgery, University of Illinois at Chicago, Chicago, IL, USA; ^2^Department of Surgery, Division of Transplant Surgery, University of Illinois at Chicago, Chicago, IL, USA; ^3^Division of Biomedical Visualization, University of Illinois at Chicago, Chicago, IL, USA; ^4^Department of Periodontics, University of Illinois at Chicago, Chicago, IL, USA; ^5^Department of Surgery, Division of Reconstructive and Plastic Surgery, University of Arizona, Tucson, AZ, USA

**Keywords:** composite tissue allotransplantation, tissue regeneration, mesenchymal stem cells, vascularized composite tissue allotransplantation, cell therapy

## Abstract

Vascularized composite tissue allotransplantation is a rapidly evolving area that has brought technological advances to the forefront of plastic surgery, hand surgery, and transplant biology. Composite tissue allografts (CTAs) may have profound functional, esthetic, and psychological benefits, but carry with them the risks of life-long immunosuppression and the inadequate abilities to monitor and prevent rejection. Allografts may suffer from additional insults further weakening their overall benefits. Changes in local blood flow, lack of fully restored neurologic function, infection, inflammation with subsequent dysregulated regenerative activity, and paucity of appropriate growth factors may all be involved in reducing the potential of CTAs and therefore serve as new therapeutic targets to improve outcomes. Strategies involving minimized immunosuppression and pro-regenerative therapy may provide a greater path to optimizing long-term CTA function. One such strategy may include mesenchymal stem cells (MSCs), which can provide unique anti-inflammatory and pro-regenerative effects. Insights gained from new studies with MSCs on composite allografts, advances in tissue regeneration reported in other MSC-based clinical studies, as well as consideration of newly described capacities of MSCs, may provide new regenerative based strategies for the care of CTAs.

## Obstacles Facing Vascularized Composite Tissue Allotransplantation

Patients receiving life-saving solid organ transplants directly contrast with those considered for vascularized composite tissue allotransplantation (VCTA). In general, VCTA recipients are physically healthy individuals except for the tissue defect; tissue transplantation is not considered life-saving or life-prolonging (1). The benefits of hand or face transplantation can include limb function as well as improvements in psychological and social well-being. A chronic immunosuppressive regimen may be needed to prevent rejection of highly antigenic tissues of multiple embryonic origins, including skin, muscles, and nerve is required (2). Rejection may not adequately present all the potential obstacles, which serve to weaken the potential of these allografts; the regenerative and repair capacity of the allograft must also be considered. Factors that impair tissue repair and regeneration in chronic wounds and therapies that modulate these responses may also be considered for composite tissue allograft (CTA) survival. Endothelial injury and corresponding changes in local blood flow, neuropathy, infection, inappropriate inflammation, and paucity of appropriate growth factors may all be involved in reducing the overall benefit of CTAs and as a consequence, serve as new therapeutic targets to improve composite allograft outcomes. While the risks of life-long immunosuppressive therapy serve as the impetus for finding alternative modulators of host immune responses to these transplanted allografts (3), focus will be directed toward regenerative and reparative strategies, including those based on mesenchymal stem cells (MSCs).

### Hand composite tissue allotransplantation

Since the first successful hand transplantation by Dubernard in 1998, over 70 hand composite tissue allotransplantations (HCTAs) ranging from wrist level to shoulder level have been performed around the world (4). The devastating loss of function after the amputation of the upper extremity makes HCTA an appealing method of restoration, as prostheses provide limited functionality. Plasticity of the human brain allows for cortical organization and adaptation after HCTA, with reversal of the cortical organization shift that occurs after sensory and motor deprivation in amputees (5). Functionality post-hand transplant can be evaluated using a 100-point functional score system, evaluating appearance (15 points), sensibility (20 points), motility (20 points), psychological and social acceptance (15 points), daily activities and work status (15 points), and patient satisfaction and general well-being (15 points) (6). In a study of 38 HCTA, all patients developed protective sensibility, 90% of patients had tactile sensibility, and approximately 80% of patients had discriminative sensation (7). Motor recovery, however, was not immediate; intrinsic muscle function was observed between 9 and 15 months post-transplantation. All patients were able to perform grasp and pinch grip, hold small objects, turn door knobs, write, and work (7). More than 85% were treated for acute rejection the first year. Correspondingly, 63.6% were diagnosed with opportunistic infections and 50% experienced metabolic complications of immunosuppression including hyperglycemia, renal dysfunction, hypertension, Cushing’s syndrome, and aseptic necrosis of the hip with bilateral replacements. It is possible that, due to the number of events occurring during the earlier period post-transplant, the mean functional scores tended to be lower in the first 4 years (range 65.5–69, single hand transplant; range 60.5–82.5 bilateral hand transplant) than the sixth and seventh years (88 points, single hand; range 84.5–94 points, bilateral hand). These findings suggest that while hand transplant provides a significant advance in regained function, its early time course is plagued with delayed function, potential tissue injury due to acute rejection, and significant complications due to chronic immunosuppression.

In terms of long-term success, there are other, less well-characterized variables which play a role. While cerebral plasticity allows adaptation to use of the hands, it does not predict psychological acceptance (8). Such acceptance may impact compliance with post-transplant medication and the success of life-long immunosuppressive therapy. Candidacy screening also presents a recognized but less discussed variable in long-term success. In the past year, in contrast to prior perception that hand transplant recipients did not experience chronic rejection (7), new evidence demonstrated, with devastating results, the effect of chronic rejection, originating with the endothelium. The amputation of the Louisville hand patient ensued after the patient had received substantive immunosuppression; Campath 1H induction (Alemtuzumab) was followed by tacrolimus and mycophenolate mofetil maintenance therapy. The patient had three episodes of acute rejection in the first 8 months characterized as a rash or slight swelling and was treated with topical tacrolimus and/or steroids. At 9 months, unmanageable ischemia secondary to severe intimal hyperplasia, confluent in the donor arteries of the allograft, resulted in amputation (9). Chronic rejection manifested as severe obliterative intimal hyperplasia of the deep vessels of the arterial tree, with the endothelium identified as a target of chronic rejection (9). The series of acute rejection preceding chronic rejection and graft loss suggests that intervention during the early course of the transplant mitigating or eliminating repetitive endothelial injury may improve long-term results. Additionally, it is plausible targeted therapeutics which provide pro-regenerative strategies for the endothelium may offset or reduce manifesting cardinal events that precipitate chronic rejection.

### Face composite tissue allotransplantation

The first partial face allotransplantation was achieved in 2005 by Dubernard in Amiens, France, and the first full face allotransplantation, containing soft tissue and bony structures, was performed in 2010 by Barret in Barcelona, Spain (10, 11). To date, over 20 facial allotransplantations with increasing comprehensiveness have been performed thus far in France, USA, Spain, and China. Face composite tissue allotransplantation (FCTA) furnishes the perfect match in facial texture, pliability, and color, as well as mimetic of function (12). While the lack of a suitable autologous substitute serves as a driving force behind FCTA, significant challenges include control of infection, prevention of rejection, psychological adaption, rehabilitation, cortical integration, and ethical practice (13).

The face plays a major role in an individual’s interaction with the outside environment. The face represents sense-of-self and identity. In addition to the senses (smelling, hearing, etc.), it conveys emotion (smiling, kissing) and plays a major role in basic physical functions (swallowing, breathing) (14). Facial disfigurement or loss of motor and sensory function has devastating psychological and social impact on an individual and FCTA manifests as a utopia for restoration (15). However, the high visibility of the face and its intimate relationship with the individual serves as a source of controversy, with ethical considerations in face donation and the donor’s family. Concerns that face transplant represents an identity exchange or that the face of a loved one would be recognizable in a stranger impart an emotive barrier to FCTA (16, 17). Related challenges arise in the recipient with psychological adaption, rehabilitation, and cortical integration.

Like hand transplant, neurologic sensory function precedes motor function. Reestablishing sensation and motion for speech, swallow, and mimicry through coaptation of the sensory and motor nerves (trigeminal and facial nerves, respectively) remain challenging. Near normal sensory recovery of the early cases has been demonstrated between 3 and 8 months postoperatively by quantitative sensory tests (18). Motor recovery has been slower with limited published objective data on motor recovery; though the first four patients were able to eat, drink, and speak within 7–10 days after transplantation (14). Functional MRI and electromyographic studies have been suggested as an objective tool to determine motor recovery in FCTA (19).

Unlike hand transplantation, facial transplantation must overcome the added hurdle of host responses directed against the transplanted mucosal barrier and associated microbiome (2, 20, 21). Much like intestinal and lung transplants which establish donor derived cellular barriers and associated microbiomes in the transplanted host, the balance between diagnosis of infection and rejection is likely to be equally problematic. So far, rates of infection compared to solid organ transplantation have been less. This observation may reflect the overall good health of the recipient prior to transplant (17), the low numbers transplanted, or an incomplete ability to differentiate infection from rejection. Alternatively, a sub-population of face transplant recipients, severely burned patients, may experience higher risk of both infection and rejection due to their proclivity for developing sepsis (17). Infection and tissue damage has been noted to associate with poorer graft outcomes (22).

Even with an ever-increasing number of FCTAs being performed, consternation still remains over the unexpected death of the world’s second face transplant recipient (China) and the death of the world’s first concomitant hand and FCTA patient (France). In 2009, Lantieri transplanted the upper 2/3 of face and bilateral hands to a 37-year-old recipient with significant burns. No acute rejection episodes occurred but the patient suffered multiresistant *Pseudomonas aeruginosa* infection on post-operative day 15 with destructive soft tissue infection, and subsequent death 65 days after transplant due to anoxic cardiac arrest from tracheostomy obstruction in the context of septic complications (23). This most recent death has spurred new questions regarding the appropriateness of concomitant face and hand tissue (CFHT) allotransplantation, stemming from length of procedure, cortical integration, antigenic load, and safety in burn patients who often undergo presensitizing events such as temporary cadaveric skin allograft coverage or blood transfusions (24, 25) and retain indolent, resistant bacteria which can reemerge in a clinically significant manner during systemic immunosuppression (26).

## Current Immunosuppressive Strategies

Original work involving cyclosporine A and successful rat hind-limb allotransplantation have paved the way for modern immunosuppressive therapy for composite tissue allotransplantation (27). Immunosuppressive protocols applied in VCTA are derived from those used in solid organ transplantation using triple-drug regimens (7). Following the guidelines established by Petruzzo et al. (7), the majority of VCTA patients began immunosuppressive induction therapy for T-cell depletion using either the polyclonal anti-thymocyte globulin (ATG) antibody or the monoclonal antibodies directed against CD25 (basiliximab) or CD52 (Campath1/alemtuzumab), followed with immunosuppressive maintenance therapy accomplished using a triple-drug cocktail of tacrolimus, mycophenolate mofetil, and steroids. Acute rejection episodes are treated with adjustment in steroid or a short course of steroid or induction agent, and use of topical tacrolimus and steroid (7). More recently, steroid reduction/avoidance and conversion of tacrolimus to sirolimus for long-term therapy (improved renal function) has been applied (28).

In addition to triple therapy, some centers have attempted novel methods to reduce immunosuppression requirements. Devauchelle and Dubernard included bone marrow donor infusion in the first case, anticipating improved survival in recipients of solid organ transplant and donor hematopoietic stem cells (29, 30). However, no benefit was seen. Hivelin et al. (31) and Lantieri et al. (23) later introduced extracorporeal photopheresis to face transplantation to reverse rejection crises by viral infection.

Decreasing the risk profile of CTA requires eliminating or reducing the obligatory life-long immunosuppression component. Induction of donor-specific immunologic tolerance has been proposed in various clinical and animal models. The Pittsburg Hand Transplant Program has found early benefit with donor bone marrow transfusion at day 15, with reduction in immunosuppressive burden (maintenance with oral tacrolimus versus triple therapy) (21) though long-term results are pending. Immunotolerance is a goal for organ and composite tissue transplantation, though particularly relevant for CTA, where procedures are not considered life-saving. Kawai demonstrated donor-specific immunotolerance across major histocompatibility complex barriers using a conditioning regimen of cyclophosphamide, anti-CD2 monoclonal antibody, thymic irradiation, and cyclosporine A before a combined bone marrow and kidney transplant in HLA haplotype mismatched living-related donor. Cyclosporine A immunosuppression was tapered over the next several months (in four of five patients) with maintenance of stable renal function without immunosuppression to date. Transient lymphohematopoietic mixed chimerism without chronic rejection was observed (32).

Progressive steps in achieving stable mixed chimerism with a non-myeloproliferative conditioning regimen and donor hematopoietic stem cell infusion (33) have been achieved in laboratory models, but clinical application is limited by the need for donor preconditioning (26). Tolerance induction through the establishment of mixed chimerism seems to require engraftment of donor hematopoietic stem cells in the recipient bone marrow compartment (26). Engrafted stem cells facilitate central and peripheral tolerance, by providing a persisting source of donor cells. Recent studies have identified novel approaches with cotransplantation of MSCs in addition to bone marrow transplantation (34) or cotransplantation of polyclonal T-regulatory cells with fully mismatched allogeneic donor bone marrow (35) to reduce the toxicity of the conditioning regimen while enhancing CTA survival. More recently, treatment with MSCs combined with preoperative irradiation and short term cyclosporine A, but without bone marrow transplantation, has contributed to prolonged composite tissue allotransplantation survival in a heterotopic hind-limb swine model (36). Current clinical translation is impeded by the lack of feasible protocols devoid of cytotoxic conditioning (e.g., irradiation and cytotoxic cells/mAbs). These treatment algorithms offer potential realization of long-term multilineage chimerism with graft tolerance.

## Mesenchymal Stem Cells in Solid Organ Transplantation

There is already strong preclinical and clinical indication for the use of MSCs in solid organ transplantation suggesting this approach could be beneficial in VCTA. In preclinical models, MSCs have not only been shown to limit the extent of injury following renal ischemia-reperfusion (37) but also demonstrated the ability to prevent rejection in a mouse model of semiallogeneic heart transplantation and a in a model of fully allogeneic islet cell transplantation (38). Additionally, MSC are capable of promoting a state of tolerance after cardiac allograft transplantation and kidney transplantation (39, 40). While there is some preclinical data suggesting that pretreatment with allogeneic MSCs may actually be detrimental to solid organ transplant by accelerating graft rejection (41), graft rejection in this study occurred at the same time when pretreated with MSCs as compared to their non-MSC-treated controls. While MSC-treated animals showed increased cellular and molecular markers for acute rejection as well a decline in functional markers, overall rejection levels, and timing were not affected by MSC pretreatment. It is unclear if this study’s findings represent unique findings or are a result of differences in immunosuppression and technical approaches as the majority or preclinical work shows great promise for MSC as a therapeutic agent. Currently, Phase I/II clinical studies are underway to determine the efficacy of MSC therapy in solid organ transplantation (42–44).

Another benefit to using MSCs is the opportunity to capitalize on the growing body of literature supporting the non-immunogenic properties of these cell populations. One key obstacle in solid organ transplantation is the need for a donor-recipient crossmatch. There is currently a large set of data suggesting that MSCs are non-immunogenic (45–47) thereby making the need for a crossmatch unnecessary. However, while these studies demonstrate the apparent immune-privileged nature of MSCs, some recent studies (48, 49) have shown the presence of anti-donor immune responses (T-cell and B-cell/antibody) following *in vivo* transplantation of allogeneic MSCs. These more recent data question both the concept that MSCs are truly immune-privileged and the reality of non-crossmatched allogeneic MSC transplantation. While it is clear that further investigation is necessary to fully understand the immunogenicity of MSCs, the potential to utilize MSCs as an “off the shelf” therapeutic agent cannot be overlooked and may offer a significant advantage in the setting of VCTA.

Because of increasing evidence to support beneficial effects of MSC, extension of MSC therapy from solid organ transplantation to VCTA is an appropriate next step for therapy in improving outcomes in CTAs. Beyond potential for facilitating immunotolerance, MSC application may potentiate therapeutic effects of repair and regeneration to those reported in acute and chronic wound models as VCTA contain skin elements, in contrast to solid organ allografts, which would benefit from accelerated closure, granulation, and angiogenesis.

## VCTA and Skin Models (Chronic Wounds, Fetal Wounds)

In VCTAs that include skin components, an effective progression through the phases of inflammation, tissue formation, and remodeling must occur since these are the overlapping phases of skin regeneration (50). Neutrophil and macrophage infiltration are necessary prerequisites to regeneration since their absence leads to deranged healing, chronic wounds with persistent inflammatory responses and associated collateral tissue destruction.

Tissue injury typically results in the secretion of several mediators of wound healing, such as platelet-derived growth factor, which attract and activate macrophages and fibroblasts (51). MSC-based therapies modulating neutrophil and macrophage responses hold potential for targeted therapies in VCTA. To date, MSC treatment of acute and chronic wounds results in more rapid epithelialization, granulation tissue formation, and angiogenesis. MSC differentiation to endothelial, keratinocyte, and pericyte cellular types in cutaneous wounds has been observed despite low engraftment efficiency (52).

The perspective of the VCTA as a chronic wound is based on similarities seen in tissue dysregulation and potential pro-regenerative synergistic targets that may act as counteragents to retarded repair mechanisms in the setting of: (1) inflammation, (2) macrophage-mediated inflammatory processes, (3) impaired epithelialization and attenuated matrix deposition, and (4) endothelial injury and intimal hyperplasia. All of these processes serve as potential targets for MSC-based therapy, due to the effect of MSCs on cytokine signaling pathways regulating immune responses and inflammation.

Mesenchymal stem cell-potentiated tissue regeneration and repair responses in chronic wound healing that may be paralleled and exploited in VCTA include: (1) MSC signaling with enhancement of cellular responses including cell survival, proliferation, migration, and gene expression; (2) MSC-conditioned media exhibiting paracrine activity as a chemoattractant recruiting macrophages and endothelial cells to the wound, including epidermal keratinocytes and dermal fibroblasts (53); (3) downstream effects of MSC signaling with reduced duration of inflammation with promotion of phagocytosis and macrophage modulation from pro-inflammatory to pro-regenerative; and (4) appropriate matrix deposition with enhanced repair and regeneration of endothelium. In this vein, MSC-based repair and regeneration in adult injury can be compared and contrasted with fetal wounding models which exhibit rapid re-epithelialization and “scarless” healing.

Fetal healing serves as an ideal model for wound repair. Pluripotent MSCs have been touted as the adult cellular proxy to reenact the tissue regenerative capacity seen early in development. Despite improved wound regeneration orchestrated by MSCs, there is no substantial evidence that MSCs promote “scarless” healing seen in fetal tissues mechanism due to MSC differentiation to replace damaged skin (52). Fetal wounds are rich in metalloproteinases and display reduced levels of transforming growth factor β1 (TGF-β1), which may serve as the basis behind scarless healing (50). Thus, many distinct signaling pathways act in concert to achieve scarless healing. MSC-based alteration of cellular activity via paracrine signaling may produce analogous effects and bear an important role in potentiating wound repair and regeneration and enhanced tissue survival. The remainder of this review will focus on aspects of MSC which can be exploited to promote long-term survival of VCTAs.

## Prospects of Mesenchymal Stem Cells in VCTA

While a great deal of research has focused on immunosuppressive strategies following VCTA, very little has focused on promoting regeneration of the allograft components. Such a strategy, in combination with immunosuppressive regimens, may improve early function and reduce immunosuppressive requirements. Induction of MSC differentiation into many of the components of VCTAs, has been reported to induce significant regenerative effects on tissues (54). However, low survival and proliferation rates of MSCs at tissue injury sites have been observed, indicating that the regenerative effects of MSCs are not derived mainly from engraftment and differentiation, but rather paracrine signaling mechanisms (52).

Since MSC-based regenerative properties are likely not due to terminal differentiation, with several studies have demonstrated that the number of MSCs administered could not numerically account for all the components of regenerating tissue (55–58) MSC-based signaling pathway modulation offers the greatest potential for enhanced regeneration and repair in VCTA (52). MSCs appear to provide their greatest regenerative effects through paracrine regulation of multiple cell types. Secreted molecules by MSCs attract macrophages, endothelial cells, keratinocytes, and dermal fibroblasts to wounds in addition to stimulating increased cellular function of these cells. This serves to not only reduce the duration of inflammation and promote phagocytosis of tissue debris, but it also induces appropriate matrix deposition, promotes the repair and regeneration of endothelium, switches macrophages from pro-inflammatory to pro-regenerative, and provide anti-microbial effects. On close evaluation of wound histology, MSCs accelerate granulation tissue formation, epithelialization, and angiogenesis (52). This enhanced tissue repair can also be seen with application of MSC preconditioned medium alone (37, 53, 59), demonstrating significant benefit from MSC-secreted signaling factors without the additive advantage of MSC pluripotency as the basis for regeneration.

Multiple strategies have been investigated to increase MSC presence at wounds, including the recruitment of endogenous MSCs as well as the direct application of MSCs to the wound. The total benefit derived from direct application of MSCs is additive with the potential for regeneration coming through both cellular engraftment and differentiation – exploiting MSC “stemness” – in addition to repair via paracrine signaling. Another strategy involves signaling endogenous MSCs to mobilize from the bone marrow and preferentially deposit in injured tissue over the surrounding, uninjured tissue (60–62). While the signaling mechanism promoting the trafficking of MSCs to skin/wounds are not fully understood at this time, chemokines SLC/CCL2 and substance P have been implicated in recruitment and circulation (52, 63, 64). These modalities of increasing MSC-potentiated effects can be paralleled into VCTA for enhanced regenerative capacity and cellular survival through improved engraftment efficiency in addition to observed paracrine-mediated responses in wound models. Overall increased potency and resilience of transplanted allografts may result from improved early graft function with MSC-enacted reduction of macrophage-derived inflammation, stimulated pro-regenerative responses with enhanced epithelialization and wound healing, and improvement in endothelium regeneration.

## Conclusion

While the risks of life-long immunosuppressive therapy serve as the impetus for finding alternative modulators of host immune responses in VCTA, MSCs may offer novel reparative and regenerative based strategies with application in these transplanted allografts. Components of tissue dysregulation involving the endothelial injury, delayed epithelialization, inappropriate inflammation, and paucity of appropriate growth factors may all be involved in reducing the overall benefit of CTAs and as a consequence, serve as novel therapeutic targets for MSCs to improve allograft outcomes.

## Conflict of Interest Statement

The authors declare that the research was conducted in the absence of any commercial or financial relationships that could be construed as a potential conflict of interest.
